# Technical and Clinical Outcomes of Embolization of Hypervascular Renal Tumors Using Tantalum-Loaded Obsidio Conformable Embolic (Ta-OCE): a single-center experience

**DOI:** 10.1007/s44343-025-00025-z

**Published:** 2025-11-17

**Authors:** Sandra Gad, Michael Mohnasky, Zachary Schrank, Bryan Harris, Priya Mody, Alexander Villalobos, Andrew Caddell, Arianna Dezfulian, Nima Kokabi

**Affiliations:** 1 Division of Vascular & Interventional Radiology, Department of Radiology, University of North Carolina at Chapel Hill, 101 Manning Drive Chapel Hill, North Carolina, NC 27514, USA.; 2 St. George’s University, West Indes, Grenada.; 3 School of Medicine, University of North Carolina at Chapel Hill, North Carolina, USA.; 4 School of Public Health, University of Washington, Seattle, USA.

**Keywords:** Renal Artery Embolization, Conformable Embolic, Renal Cell Carcinoma, Angiomyolipoma

## Abstract

**Purpose:**

To evaluate the efficacy and safety of renal artery embolization (RAE) in hypervascular renal tumors using Tantalum-Loaded Obsidio^™^ Conformable Embolic (Ta-OCE).

**Methods:**

A single-center institutional review board (IRB) approved retrospective analysis of consecutive patients with benign and malignant hypervascular renal tumors treated with Ta-OCE between May 2023 and June 2024 was performed. Specific clinical data on tumor size reduction, renal function preservation, bleeding, and complications were collected. Follow-up imaging was conducted using MRI or CT scans. Adverse events were evaluated according to the Common Toxicity Criteria for Adverse Events (v.5). Student’s t-test was used to compare continuous variables.

**Results:**

Overall, *N* = 10 patients (mean age 64.1 + 14.9 (SD) years, 46% female) were included; 5 patients with renal cell carcinoma (RCC) and 5 patients with angiomyolipoma (AML). Technical success was achieved in all 10 patients. There was a significant reduction in tumor diameter of 1.03 cm following embolization (*p* = 0.0009). There was no significant change in renal function post-RAE at 1 month, GFR pre-(72.8 ± 23.5 mL/min/1.73 m^2^, GFR post 63 ± 24.7 mL/min/1.73 m^2^, with a mean change in GFR (%) of − 13.5 ± 11.2%. (*p* = 0.116). Additionally, no significant bleeding requiring further intervention was experienced. One patient experienced a Grade 1 adverse effect due to an underlying heart condition.

**Conclusion:**

Embolization of hypervascular renal tumors with Ta-OCE appears to be safe and effective. Prospective, ideally multicenter studies are needed to further clarify the ideal scenarios for the use of Ta-OCE in hypervascular renal tumors.

## Graphical Abstract

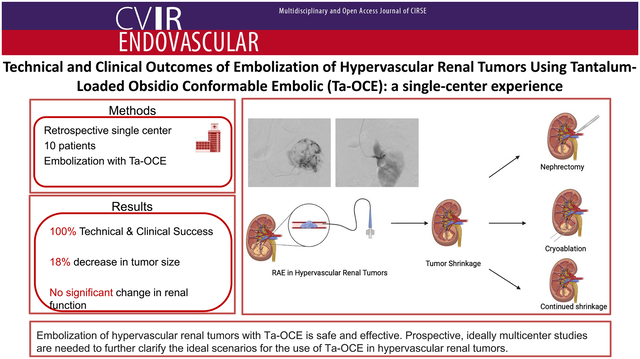

## Introduction

Hypervascular renal tumors include several etiologies, with renal cell carcinoma (RCC) being the most common malignant renal tumor, and angiomyolipoma (AML) as the most common benign renal tumor [1]. Hypervascular renal tumors present a significant clinical challenge, largely due to their propensity for spontaneous retroperitoneal bleeding. This bleeding risk often requires urgent intervention to prevent hemorrhagic complications, which can be life-threatening if not promptly managed [2, 3]. Current management guidelines include active surveillance, renal artery embolization (RAE), thermal ablation, or surgical management, which is usually dependent on the tumor’s size, vascularity, and patient-related factors [2–4].

As a minimally invasive alternative to surgery, RAE has gained traction due to its potential to achieve tumor shrinkage through ischemia and reduce the risk of hemorrhage. Specifically pertaining to RCC, thermal ablation (TA) is considered an alternative treatment to partial nephrectomy for localized small renal tumors by American Urological Association [5]. Although, historically TA was reserved for tumors < 3 cm localized to the kidney (T1aN0M0), more contemporary data has shown the efficacy of TA in tumors up to 7 cm (T1bN0M0) [6]. RAE was first described in 1973, has evolved significantly, particularly in the management of hypervascular renal tumors [7, 8]. RAE is commonly utilized in patients with larger renal tumors undergoing TA to reduce the risk of intraprocedural bleeding, tumor seeding, and to reduce the size of the tumor prior to embolization [9].

Standardized recommendations regarding embolic use for hypervascularized renal tumors are currently lacking. This gap has resulted in diverse approaches in clinical practice, with several reports utilizing a range of embolic agents, including coils, ethanol, lipiodol, liquid embolics such as ethylene vinyl alcohol (EVOH) copolymer, and N-butyl cyanoacrylate (NBCA) glue and/or particles [3, 10]. Each of the aforementioned embolic agents present unique advantages and limitations based on the patient’s clinical context. Furthermore, ongoing advancements continue to drive the development of novel embolic materials.

One such embolic product was recently developed for hypervascular tumors. Obsidio ^®^ (Boston Scientific, Marlborough, MA) is a novel tantalum-loaded nanocomposite hydrogel embolic (Ta-OCE) that conforms to the target vessel’s shape and provides mechanical occlusion. Currently, Tantalum-loaded Ta-OCE Conformable Embolic (Ta-OCE) is FDA 510 k cleared for embolization of hypervascular tumors and bleeding in peripheral vessels with diameters < = 3 mm [11].

Limited clinical data is available regarding this embolic’s effectiveness and complication profile in hypervascular tumors and in particular renal ones [12]. Early reports suggest potential benefits, such as ease of delivery and minimal off-target embolization [13]. Given the limitations of conventional embolic agents, such as prolonged preparation time, Computed Tomography (CT) artifacts, catheterization, and reliance on coagulation with particles or coils, this study aims to evaluate the technical success, short-term safety, and effectiveness of hypervascular renal tumors embolized with Ta-OCE.

## Materials and methods

### Study design and patient selection

A retrospective review of consecutive patients with hypervascular renal tumors who underwent selective RAE with Ta-OCE between July 2023 and July 2024 at our institution was performed. This is a Health Insurance Portability and Accountability Act (HIPAA)-compliant study, which was approved by the Institutional Review Board (IRB). A total of ten patients (*N* = 10) underwent RAE at our institution (mean age 64.1 + 14.9 (SD) years, 46% female) (Table 1).

### Renal artery embolization

**Fig. 1:**
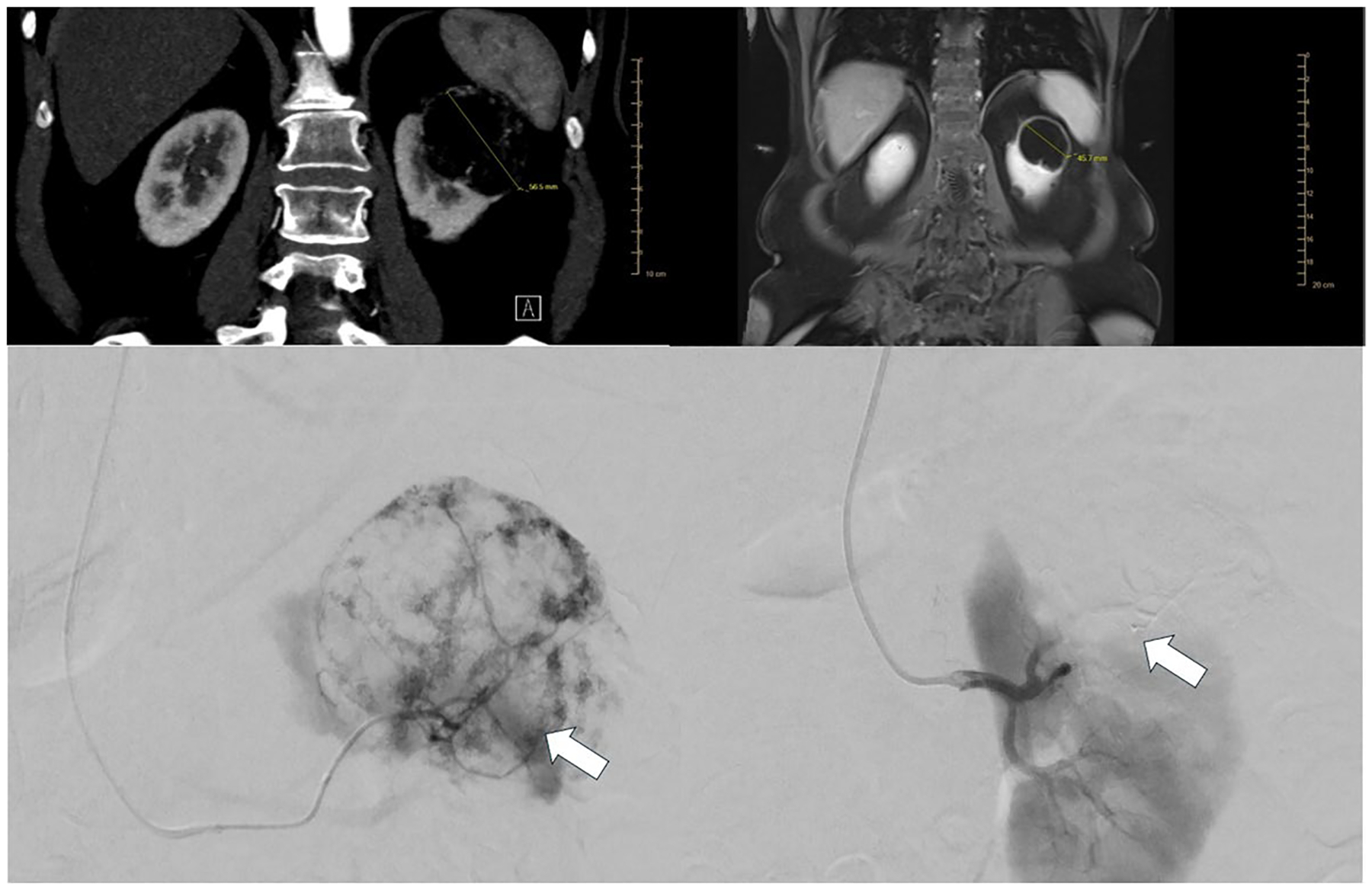
A 65-year-old female renal AML (**A**) Coronal contrast-enhanced CT scan shows a 5.7-cm enhancing renal mass in the upper pole of the left kidney (**B**) MRI at 3 months post-embolization demonstrating tumor diameter reduction of 1 cm. **C** Selective left renal arteriogram demonstrating a large hypervascular renal cell carcinoma (arrow) before embolization. **D** Microcatheter-mediated embolization of the hypervascular mass using Ta-OCE. Note the decreased regions of tumor vascularity (arrow)

**Fig. 2:**
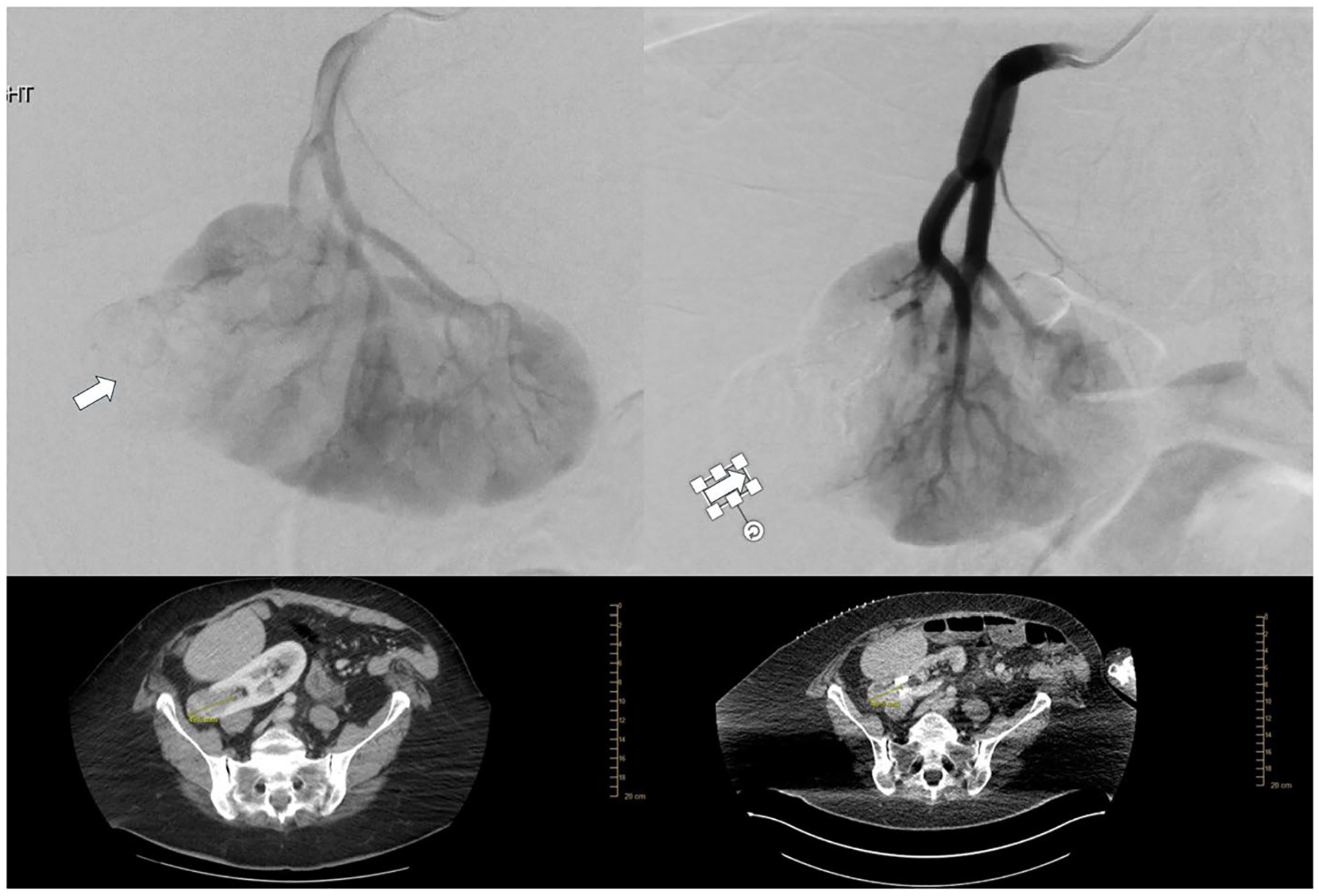
A 65-year-old female with RCC, who failed partial nephrectomy due to adhesions, was referred to IR for thermal ablation. **A** Selective left renal arteriogram demonstrating a large hypervascular renal cell carcinoma (RCC) before embolization (arrow). **B** Microcatheter-mediated embolization of the hypervascular mass using Ta-OCE. Note the decreased regions of tumor vascularity (arrow). **C** Axial CT of the RCC measuring 4.9 cm **D** Follow-up axial CT one-month post-embolization prior to cryoablation, shows approximately 18% reduction in the diameter of the renal lesion. Note Ta-OCE limited artifact

Moderate sedation was used for all embolization procedures. In all ten patients, selective RAE was performed with a coaxial 2.0 Fr microcatheter (Truselect, Boston Scientific, Marlborough, MA) through a 5 Fr base catheter from a radial or femoral arterial approach. After selecting the main renal artery with the base catheter, digital subtraction angiography was performed to evaluate and detect the supplying vessels of the target tumor (Figs. 1 and 2). 3D cone beam computed tomography (3D-CBCT) was then performed to ensure complete targeting of tumors.

The tumor-feeding arteries were catheterized with a 2.0 Fr Truselect microcatheter (Boston Scientific, Marlborough, MA). Embolization was then performed under fluoroscopy using Ta-OCE. The embolic material was injected until the complete casting of the feeding artery and the stasis of embolic flow at the tip of the microcatheter. If more than one artery needed to be embolized, the microcatheter was removed and residual Ta-OCE in the microcatheter was flushed out. Then, using the same technique described above, other target vessel(s) were selected with the microcatheter, and embolization was performed. At the conclusion of the embolization(s), angiography through the based catheter in the renal artery was performed to ensure non-opacification of target tumor (Figs. 1 and 2).

### Renal function assessment and imaging follow-up

Following embolization, subjects were evaluated for renal function using an estimated glomerular filtration rate, and the percentage change in GFR was calculated.

Follow-up imaging contrast-enhanced CT and/or MR imaging was obtained at approximately 1 to 3 months post-embolization to evaluate therapeutic response and tumor shrinkage for the benign tumors. Tumor size reduction was assessed descriptively by comparing maximal tumor diameter on follow-up contrast-enhanced CT or MRI with baseline pre-embolization imaging. No predefined response threshold was used, given the exploratory nature and small sample size, percentage changes are reported. When feasible, the same modality used at baseline was used for follow-up measurements to maintain consistency.

### Cryoablation

Patients with RCC underwent cryoablation 1–2 months post-embolization. The interval between embolization and cryoablation was intentionally set at approximately 4–8 weeks to ensure complete devascularization and decrease the risk of hemorrhage during cryoablation. This staged approach was selected to optimize ablation efficacy and procedural safety, particularly for larger vascular tumors in our cohort. Cryoablation was performed percutaneously under CT guidance. Two to four probes were carefully inserted into the renal mass, and the probe tips were positioned in firm contact with the target lesion for stability. A 10-min freeze, 5-min thaw, and 10-min refreeze cycle was applied, achieving intratumoral temperatures of approximately − 40 °C. Intermittent CT imaging was performed during each freeze cycle to ensure complete ice-ball coverage of the tumor and to monitor the relationship of the ablation zone to adjacent organs. Following ablation, the probes were gradually warmed to 20 °C before removal. A post-procedural CT scan was obtained to assess for complete ablation and assess for complications. The size of the tumor on planning contrast-enhanced CT scan during cryoablation was compared to the size of the tumor on CBCT pre-embolization. In addition, imaging was used to assess for target vessel recanalization, which was defined as recurrent opacification of the embolized target feeding vessel of the tumor.

### Efficacy

Technical success was defined as the complete occlusion of target vessels on the final renal angiogram. Clinical success was defined as the absence of bleeding after embolization and shrinkage of tumor size post-embolization. At the time of follow-up, renal function was assessed by glomerular filtration rate (GFR) and compared with renal function before embolization. The percentage change in GFR was also reported. Complications that occurred during and after the procedure were evaluated and categorized according to the Common Terminology Criteria for Adverse Events, version 5.0. (CTCAE v 5.0).

### Statistical analyses

Continuous variables are expressed as mean and SD. Categorical variables are expressed as frequency and proportion. The glomerular filtration rate was compared before and after embolization using a paired t-test. All statistical analyses were performed using R software version 4.2.3 (R Foundation for Statistical Computing, Vienna, Austria).

## Results

A total, 13 arteries were embolized. The median volume of Ta-OCE used was 0.25 mL (range, 0.10–0.40 mL), and the median fluoroscopy time was 16 min (range, 7.3–89 min) (Table 2).

Technical success was 100%. Specifically, there was complete occlusion of target vessels on the final renal angiogram. Among the 5 patients treated for AML, 4 patients had an idiopathic etiology, while 1 was associated with underlying tuberous sclerosis. For the AML embolization (*n* = 5), at 3 months post-embolization, all patients had complete devascularization of tumors on MRI.

Indications varied for the five patients treated for renal cell carcinoma (RCC) (*n* = 5). One patient underwent embolization due to persistent hematuria from renal mass, another required embolization prior to nephrectomy as a palliative measure, and finally, three underwent embolization prior to cryoablation. In the patients that underwent subsequent cryoablation, the mean period from RAE to ablation was 25.5 days (range:18–23).

All tumors had decreased in size with a mean maximum dimension of 4.8 cm (range: 1.4–9.3) (Table 2).

The procedure was well-tolerated overall, with only one serious adverse event (AE) reported. One patient experienced intraoperative shortness of breath, which was managed with supplemental oxygen. The event was attributed to volume overload in the context of preexisting heart failure and was deemed to be unrelated to embolization. No long-term sequelae were observed.

Embolization was effective after 1 trial of embolization with an overall treatment effectiveness rate of 100%. Follow-up imaging in 9 of the patients at 56 days (range, 18–90 days) demonstrated durable occlusion with no target vessel recanalization. One patient underwent nephrectomy for definitive treatment. All nine tumors exhibited positive response outcomes, with a significant reduction in tumor diameter of 1.03 cm (*p* = 0.0009) following embolization. Renal function information was available in a subset of 8 patients (80%). The mean glomerular filtration rate (GFR) decreased from 72.8 ± 23.5 to 63 ± 24.7 mL/min/1.73 m^2^ following embolization, with a mean change in GFR (%) of − 13.5 ± 11.2%. However, this decline was not statistically significant (*p* = 0.116) (Table 2).

## Discussion

This single-center experience demonstrated 100% technical success, a mean 21% tumor diameter reduction, and preserved renal function following Ta-OCE embolization of hypervascular renal tumors. These results indicate that Ta-OCE can achieve effective and selective devascularization comparable to established embolic agents while maintaining a comparable safety profile.

Preoperative embolization has shown several benefits, including decreased intraoperative blood loss, low surgical complication rates post-surgery, and decreased mortality rate [7, 8]. A study by Zielinski et al. demonstrated a survival advantage in patients receiving RAE pre-surgery compared to surgery alone [14]. The RAE application has been extended to palliative patients with unresectable RCC for symptom management and/or survival benefit. A retrospective analysis of patients with non-resectable RCC compared 24 patients who received RAE with ethanol to 30 patients who did not receive RAE and reported a significantly increased survival rate in the RAE group, where the median survival was significantly longer in the TAE group (229 days) compared to the non-TAE group (116 days). Furthermore, the 1-, 2-, and 3-year survival rates were higher for patients undergoing TAE (29%, 15%, and 10%, respectively) compared to those who did not undergo the procedure (13%, 7%, and 3%, respectively) [15].

Traditional embolics used for hypervascular renal tumors have shown marginal occlusion ability and often require large vessel occlusion with coils [16, 17]. A recent retrospective study that used particles, coils, or a combination of both reported only 50% technical success, and complete devascularization was seen in only 50% of their cohort [4]. In contrast, our study achieved a technical success rate of 100%, with reduced/absence of vascular enhancement of tumors in all patients.

All patients with follow-up imaging (*n* = 9) in this cohort demonstrated a 21% reduction in tumor diameter at follow-up. This finding is consistent with a recent study using PVA particles, microcoils, ethanol (with or without lipiodol), or a combination of these in patients with angiomyolipomas (AMLs), which reported a reduction of 23% at 3 months and 30% at 1 year [4]. Given AMLs’ propensity for gradual size reduction post-embolization, further longitudinal studies would be valuable to evaluate long-term tumor shrinkage with Ta-OCE [18].

The benefits of sequential renal artery embolization and cryoablation have been previously reported for patients with RCC undergoing cryoablation [19, 20]. Benefits include a decreased risk of renal collecting system injury and a lower complication profile due to the decreased number of probes needed to treat the lesion [19, 20]. Many retrospective studies reported promising technical outcomes, and clinical outcomes [19, 20]. Ongoing clinical trials such as the EMBARC multicenter single-arm prospective trial results are highly anticipated, providing a better understanding of neoadjuvant RAE [21].

Renal function is a critical consideration, given the “end artery” anatomy of the renal vasculature, which lacks substantial intrarenal collaterals and increases the potential for damage to normal renal parenchyma post-embolization [22]. In our cohort, patients experienced no effect on renal function, congruent with reported results when using EVOH copolymer [23]. The ability to deliver the Ta-OCE selectively in the targeted tumors with minimal surrounding renal parenchyma sacrifice is the likely explanation for the renal function preservation observed in this study.

Numerous embolic agents with varying characteristics are currently available on the market for transcatheter embolization. Particles, ethanol, and iodized oil mixture are the most commonly used embolic for RAE [24]. However, there is a paucity of comparative data for embolic. In a series of 45 patients with renal bleeding lesions, coils, NBCA glue, and PVA particles reported no significant difference among coil, NBCA glue, and PVA [25]. They reported primary clinical success in 91% and secondary success in 92% after re-embolization, with post-embolization syndrome in 31% of cases and non-target embolization in 2 cases, using glue and PVA [25]. There are no standard recommendations regarding embolic use for hypervascular tumors available. The choice of embolic agent often depends on the physician’s preference and desired outcomes. One desired outcome for hypervascular tumors is ischemia, which can lead the tumor shrinkage. This ischemic effect can be effectively induced with a range of embolic agents.

Ta-OCE offers several unique advantages that combine the benefits of multiple embolics in a single formulation. Ta-OCE is considered a conformable, non-adhesive, and permanent embolic agent [11]. Overall, Ta-OCE’s mechanically occlusive but nonadhesive nature makes it ideal for preoperative embolization, allowing for effective vessel occlusion without compromising surgical field visibility.

Ta-OCE’s ready-to-use formulation is convenient for both elective and emergent settings, eliminating the preparation time required for other liquid embolic agents, such as glue, which must be mixed with ethiodized oil or EVOH copolymer, which needs to be shaken for 20 min to ensure homogeneity [8, 22]. While glue has shown excellent technical and clinical success rates, major drawbacks with glue include uncontrolled release, high risk of migration or non-targeted embolization, microcatheter entrapment because of short polymerization time, and a more challenging learning curve for the user [11].

Ta-OCE has the advantage of being effective regardless of the patient’s current coagulopathy status, which is a limitation of coils and particles that rely on the patient’s coagulopathic status to induce thrombosis [10]. In our cohort, three patients had low platelets, and no rebleeding events occurred post-embolization. Furthermore, Ta-OCE requires only 0.2 ml for complete occlusion in cases where multiple coils or in conjunction with other embolics such as glue would otherwise be necessary [26].

In addition to its ease of use, Ta-OCE’s rapid solidification time within seconds offers a distinct advantage over EVOH copolymers, which require a slower polymerization process time [10]. The shear-thinning properties of Ta-OCE further enhance its utility by allowing precise injection at the microcatheter tip, minimizing the risk of reflux—a common concern with NBCA glue, as previously eluded to [13]. Ta-OCE’s compatibility with a variety of microcatheters increases its applicability across clinical settings, adapting to operator preferences and institutional resources. Finally, Ta-OCE results in minimal beam hardening artifacts in conventional computed tomography (CT) and cone-beam CT (CBCT) compared to other embolic such as EVOH copolymer, glue and coils [10]. Embolization-related artifacts can represent a significant obstacle in the detection of hemorrhage during or post-embolization on CT [27]. The decreased tantalum concentration in Ta-OCE results in decreased imaging artifact.

In terms of patient experience, our study cohort reported minimal intra- and post-procedural pain, compared with the discomfort associated with agents like EVOH copolymer and particles. This may be attributed to the biocompatibility of Ta-OCE.

Our results are consistent with reported brief reports using Ta-OCE that included hypervascular tumors. A recent brief report included acute tumor bleeding (*n* = 3) that was occluded effectively using Ta-OCE with no adverse effect, as well as the use of Ta-OCE for hematuria due to RCC (*n* = 1) [13]. More recently, a study involving hypervascular tumor embolizations included 9 renal angiomyolipomas, 2 primary renal cell carcinomas, and 2 RCC metastases, reporting 100% technical success with no migration on CT. However, the effectiveness of this embolic was not reported [28]. In a brief report using Ta-OCE, six renal arteries were treated, with one requiring re-intervention; however, the target arteries had been previously embolized with microspheres. Ta-OCE was used in conjunction with coils to achieve definitive hemostasis [29].

This study presents several limitations, including a small sample size and a retrospective nature. Second, the nonrandomized design is not well-suited for comparing Ta-OCE to other conventional embolic agents in terms of technical and clinical successes, making it difficult to draw relative advantages. Third, potential biases when interpreting target vessel recanalization, due to a lack of quantitative perfusion analysis or blinded review, which may introduce detection and observer bias. Fourth, this study reflects a single-center experience with renal embolization using Ta-OCE, which limits its generalizability. Finally, there was a lack of cost analysis, as the financial implications of using Ta-OCE relative to more conventional agents, such as coils, particles and glue. Further studies should evaluate the cost-effectiveness of Ta-OCE in reducing adjunct healthcare expenses, such as decreased hospitalizations due to rebleeding or additional interventions.

## Conclusion

In this small retrospective series, Ta-OCE embolization achieved complete angiographic devascularization, resulting in significant tumor size reduction and preservation of renal function. These findings suggest that Ta-OCE might be a promising embolic in the embolization of hypervascular renal tumors. However, a prospective, multicenter experience is warranted to further establish its clinical benefits.

## Figures and Tables

**Table 1:** Patient characteristics

Characteristic		n = 10
Sex	Male	2 (20%)
	Female	8 (80%)
	Mean Age ± SD (yr)	64.1 ± 14.9
Ethnicity	African American	2
	White	7
	Hispanic	1
Renal Tumor	Angiolipoma	5 (50%)
	Renal Cell Carcinoma	5(50%)
Indications	Interval growth for AML	5(50%)
	RAE prior to cryoablation	3 (30%)
	Hematuria	1 (10%)
	RAE prior to nephrectomy	1 (10%)

**Table 2:** Summary of outcome data

Arteries embolized (n)	13	
Mean arteries per patient (n)	1.3 ± 0.5	
Follow-up duration (days)	56 ± 18	
Embolization to cryoablation (days)	25.5 ± 3.0	
Kidney Function	Pre embolization eGFR (mL/min/173 m^2^)	72.8 ± 23.5
	Post embolization eGFR (mL/min/173 m^2^)	63.0 ± 24.7
	Change in eGFR %	13.5 ± 4.2
Tumor Size	Pre embolization (cm)	4.8 + 2.6
	Post Embolization (cm)	3.8 + 2.2
	Mean percent change in diameter (%)	21 + 5.0

## Data Availability

The datasets used and/or analyzed during the current study are available from the corresponding author on reasonable request.

## Abbreviations

RCC: Renal Cell Carcinoma
AML: Angiomyolipoma
RAE: Renal artery embolization (RAE)
Ta-OCE: Tantulum Loaded Obsidio^™^ Conformable Embolic
CT: Computed Tomography
CBCT: Cone-Beam CT
TA: Thermal Ablation
